# Impulsivity is associated with suicide attempt in mood disorders with complex moderation by diagnosis and subjective social status

**DOI:** 10.21203/rs.3.rs-8970654/v1

**Published:** 2026-04-28

**Authors:** Pooya Hazegh, Gail Harmata, John Barsotti, Jess G. Fiedorowicz, Aislinn Williams, Jenny Gringer Richards, Aubrey Chan, Susan Shen, Jamini Bhagu, John Wemmie, Vincent Magnotta

**Affiliations:** University of Iowa; University of Iowa; University of Ottawa; University of Iowa; University of Iowa; University of Iowa; University of Iowa; University of Iowa; University of Iowa; University of Iowa

**Keywords:** Mood disorders, Bipolar disorder, Major depressive disorder, Impulsivity, Suicide attempt, S-UPPS-P

## Abstract

**Aims:**

Suicide Attempt (SA) is common among individuals with mood disorders, and trait impulsivity is a strongly associated risk factor. However, findings on the relationship between trait impulsivity and SA vary widely. We hypothesized that impulsivity is associated with SA in patients with mood disorders (either MDD or BD), and we tested how diagnostic category and Subjective Social Status (SSS) may impact this relationship.

**Methods:**

Participants with bipolar disorder (BD; types I and II) and major depressive disorder (MDD) were assessed at intake in a cross-sectional analysis. Impulsivity was measured with the Short UPPS-P Impulsive Behavior Scale (S-UPPS-P), which assesses five facets of impulsivity (negative urgency, positive urgency, premeditation, perseverance and sensation seeking). SA history was determined using the Columbia-Suicide Severity Rating Scale (C-SSRS). The sample included 153 participants (74 BD, 79 MDD), of whom 66 had at least one prior suicide attempt. Logistic regression analyses examined associations between impulsivity scores and prior SA, controlling for age and sex. We also tested how controlling for diagnosis and SSS affected the model, and whether diagnosis and SSS moderated the relationship between impulsivity and attempt.

**Results:**

S-UPPS-P total score and the subscales negative urgency and positive urgency were associated with prior suicide attempt across the sample. S-UPPS-P total score was no longer significant when adjusting for diagnosis and SSS. When testing how these three variables may interact, we found a significant three-way interaction between S-UPPS-P total score, diagnosis, and SSS.

**Conclusion:**

The relationship between impulsivity and suicide attempt is complex and moderated by diagnosis and SSS.

## 1. Introduction

Mood disorders are strongly associated with elevated rate of suicide attempt (SA) and death by suicide [1–3]. Suicide Attempt is a potentially self-injurious behavior with a nonfatal outcome, for which there is evidence (either explicit or implicit) that the person intended at some (nonzero) level to kill himself/herself. A suicide attempt may or may not result in injuries [4]. Estimates of the lifetime SA rate in bipolar disorder (BD) range from 20% to 56%, which is approximately 15 times higher than in the general population [5–6]. In individuals with major depressive disorder (MDD), the lifetime SA rate is estimated to be around 31% [7]. Beyond diagnosis, additional factors play an important role in the high rate of SA in mood disorders. Impulsivity has been suggested as one such risk factor and the American Association of Suicidology recognizes impulsivity as both a chronic and an acute risk factor for suicide [6–8]. Elevated impulsivity is linked to the transition from suicidal thoughts to suicide attempts [9–10].

Impulsivity is defined as a predisposition toward rapid, unplanned reactions to internal or external stimuli, without regard to the potential negative consequences [11]. Impulsivity has also been conceptualized as a cognitive dimension reflecting a lack of planning and concern about future outcomes [12], which results in poorly conceived and hasty actions, and may lead to undesired outcomes [13]. Impulsivity is typically divided into two components: state and trait impulsivity. State impulsivity is the moment-to-moment experience of impulsivity [14] and is modulated by affective state [15] and autonomic reactivity [16]. Laboratory-based cognitive tests have been designed to measure state impulsivity through the assessment of immediate actions and decisions [17] using such tests as the Immediate Memory Task-Delayed Memory Task [12]. Trait impulsivity, conversely, remains more consistent over time and reflects one’s personal characteristics [16–18]. Self-report questionnaires such as the Barratt Impulsiveness Scale (BIS) and Negative Urgency-Premeditation-Perseverance-Sensation Seeking-Positive Urgency Scale (UPPS-P) [19–20] are commonly used to assess trait impulsivity [19–20]. One prominent model of impulsive traits argues that impulsivity has four distinct impulsigenic traits: a lack of tendency to think and plan (lack of planning or premeditation), difficulty persisting towards goals (lack of perseverance), act on impulses in the face of negative emotions (negative urgency), and engage in thrill-seeking behaviors (sensation seeking) [14],[21]. Later, Cyders et al. proposed the existence of *positive urgency*, or the tendency to engage in rash actions when in an unusually positive mood [22]. Nearly all global self-report measures of trait impulsivity, including S-UPPS-P, reflect some combination of these five impulsive traits [14].

Beyond its association with SA, trait impulsivity has been found to be elevated in individuals with BD, regardless of the phase of the illness [23–26]. Trait-impulsivity has also been found to be elevated in the unaffected siblings of individuals with BD, suggesting a genetic contribution to impulsivity [27]. Multiple studies have also explored the relationship between MDD and different aspects of trait impulsivity. While some studies have reported elevated self-reported trait impulsivity in individuals with MDD [28–31], others have not [32–35]. These inconsistencies highlight the need for further research to clarify the role of trait impulsivity in mood disorders and its contribution to SA.

Subjective social status (SSS), defined as an individual’s perception of their position within the social hierarchy [36], has become an important focus in recent research. The Social Rank Theory (SRT), which emphasizes the role of SSS, suggests that perceived social standing exerts a pervasive influence on depression and SA [37]. According to SRT, individuals with higher SSS typically have greater access to financial and material resources and enjoy stronger social reputations, whereas those with lower SSS may experience material deprivation and a diminished sense of internal locus of control—factors linked to negative affect, mood disorders, and suicidality [38–39]. Beyond its association with mood disorders, SSS has also been identified as a reliable predictor of trait impulsivity [40]. Furthermore, low SSS is associated with disorders characterized by impulsivity, such as substance use disorders [40–41] and emotional eating [42]. These findings underscore the need for further research to clarify the complex interplay between impulsivity, SSS, and suicide.

Many studies examining the relationship between suicide attempt and impulsivity in patients with mood disorders have treated MDD and BD as distinct diagnostic categories [43–46], yielding mixed results. However, some researchers propose that major mood disorders may be better conceptualized as existing along a continuum [47–48],[13]. This perspective challenges the unipolar-bipolar dichotomy and supports a broader conceptualization of bipolarity, echoing Kraepelin’s original vision of the manic-depressive continuum. Within this framework, mood disorders would be a part of the so-called “broad bipolar spectrum” [49]. Determining which conceptualization provides greater accuracy in research and clinical practice remains an important area for future investigation.

Based on these premises, the present cross-sectional study aimed to investigate the relationship between trait impulsivity score and SA in an outpatient sample of participants with mood disorders (BP and MDD). We hypothesized that higher levels of trait impulsivity would be associated with greater likelihood of past SA in patients with mood disorders. We also examined how adjusting for diagnosis and subjective social status (SSS) affected this relationship, and whether diagnosis and SSS moderated the association between impulsivity and prior SA.

## 2. METHODS

After receiving institutional review board (IRB) approval from the University of Iowa, individuals with BD (type I and II), MDD, and frequency matched controls (based on age, gender, and SSS) were recruited into this study. The present study uses assessment data from a larger neuroimaging study of suicidal behavior where the analysis was focused on those participants with a mood disorder. Potential participants were screened and excluded from the study for comorbid neurological disorders, past loss of consciousness for more than 10 minutes, comorbid primary psychotic disorders, current alcohol or substance use disorder, current pregnancy, and MRI contraindications. After screening for eligibility, participants and voluntarily provided informed written consent before enrolling in the study. At the time of this analysis, there were 79 participants with MDD and 74 participants with BD (70 participants with BD type I and 4 participants with BD type II) enrolled into the study.

### 2.1 Subject Assessment

All participants were evaluated using the Structured Clinical Interview for Diagnostic and Statistical Manual of Mental Disorders V Research Version (SCID-V-RV). Participants with a diagnosis of BD (types I and II) and MDD based on the SCID-V were included in this analysis. To assess impulsivity, we used the short form of UPPS-P (S-UPPS-P) Impulsive Behavior Scale, which consists of 20 questions [50]. The short form was chosen to reduce participant burden; it has high internal consistency (0.74–0.88 across subscales) despite containing only one-third of the full UPPS-P [50] scale. The S-UPPS-P has five subscales (comprised of four questions each) including: 1) negative urgency: the tendency to act rashly while in an intense negative mood; 2) lack of premeditation: the tendency to not take into account the consequences of actions; 3) lack of perseverance: the tendency to have difficulty staying focused on a task that can be long, boring or difficult; 4) sensation seeking: the tendency to seek out novel and thrilling experiences; and 5) positive urgency: the tendency to act rashly while in an intense positive mood. Each question is scored from 1 to 4, resulting in the total score ranging from 20 to 80, with higher values indicating higher impulsivity traits [51].

Several other assessments were obtained from the study participants, including the following: the Columbia-Suicide Severity Rating Scale (CSSR-S) [52], the MacArthur Scale of subjective social status (SSS) [53], the Montgomery-Åsberg Depression Rating Scale (MADRS) [54], the Young Mania Rating Scale (YMRS) [55] and the Adverse Childhood Experiences Scale (ACE) [56]. The participants also provided a list of current medications.

### 2.2 Data Analysis

Basic study demographics (age, sex, SSS, education history, ACE, MADRS and YMRS) were compared between the diagnostic categories (BD and MDD) as well as between SA status (Attempt + and Attempt−). In comparing demographics between groups, a Chi-Square test of independence was used for sex, diagnosis, and SA status while all other comparisons used a Student’s t-test. In addition, Spearman’s rank correlation analysis was used to assess the relationship between the potential covariates: age, sex, SSS, and diagnosis (MDD or BD). None of the variables were highly correlated, with correlation coefficients ranging from − 0.30 to 0.28. Therefore, we determined that we could utilize all of the variables as potential covariates in our analyses with low risk for multicollinearity. We then proceeded to assess the relationship between impulsivity (independent variable) and SA history (dependent variable) using a logistic regression model in a tiered approach.

In tier 1, the logistic regression model controlled for age and sex.


$$\text{logit}(\text{p})=\beta_{0}+\beta_{1}\;\text{Impulsivity}+\beta_{2}\;\text{Age}+\beta_{3}\;\text{Sex}$$

In tier 2, SSS was added to the statistical model.


$$\text{logit}(\text{p})=\beta_{0}+\beta_{1}\;\text{Impulsivity}+\beta_{2}\;\text{Age}+\beta_{3}\;\text{Sex}+\beta_{4}\;\text{SSS}$$

Finally, in tier 3, a three-way, fully-crossed interaction between diagnosis, SSS, and impulsivity was used.


$$\text{logit}(\text{p})=\beta_{0}+\beta_{1}\;\text{Impulsivity}+\beta_{2}\;\text{Age}+\beta_{3}\;\text{Sex}+\beta_{4}\;\text{SSS}+\beta_{5}\;\text{Diagnosis}+\beta_{6}\;(\text{Impulsivity}\times \text{SSS})+\;\beta_{7}\;(\text{Impulsivity}\times \text{Diagnosis})+\beta_{8}\;(\text{SSS}\times \text{Diagnosis})+\beta_{9}\;(\text{Impulsivity}\times \text{Diagnosis}\times \text{SSS)}$$

In all three tiers, separate models were employed for the S-UPPS-P total score and each of the 5 subscales. Statistical significance for the study was set to p < 0.05, without correction for multiple comparisons since the five sub-scales contributed to the overall total impulsivity score. Given the small number of BD type II cases (n = 4), we repeated all analyses after removing these participants.

All statistical analyses were conducted in R version 4.3.1 (R Core Team, 2023) and RStudio (RStudio Team, Version 2023.03.0 + 386) with the addition of the following packages: Car [57], corrplot [58], dplyr [59], ggplot2 [60], jtools [61], readxl [62], plyr [63], skimr [64].

## 3. Results

### 3.1 Demographics

Demographics of the participants are shown in Table 1 and **Supplemental Table 1**. The participants with a prior suicide attempt were not significantly different in sex, age, and years of education as compared to those without an attempt. However, those with a prior attempt had significantly higher depressive (MADRS 17.5 vs. 12.2, *p* = 0.001) and manic symptoms (YMRS 7.4 vs. 3.6, *p* = 0.002). In addition, participants with a prior SA had lower SSS (SSS 4.9 vs. 5.7, *p* < 0.001) and a higher number of ACE (ACE 4.2 vs. 2.5, *p* = 0.001) as compared to those without a prior attempt. Finally, the participants with a prior suicide attempt were more likely to have a diagnosis of BD as compared to MDD (61% vs. 39%, *p* = 0.013) with approximately half of the participants with BD reporting a prior SA while only a third reporting an attempt in the MDD group.

### 3.2 Impulsivity and SA

**Figure 1:**
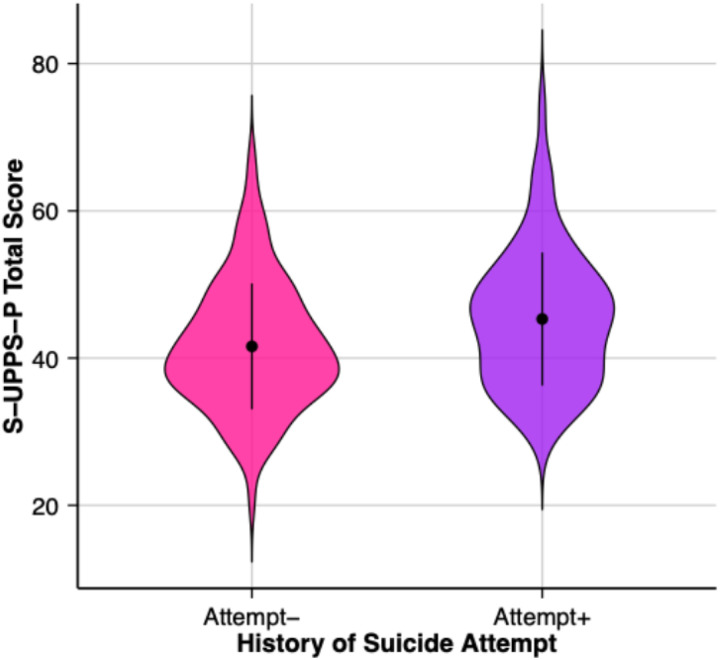
Distribution of total impulsivity scores across suicide status groups. S-UPPS-P = Short UPPS-P Impulsive Behavior Scale.

The tier 1 analysis (Table 2) explored the relationship between impulsivity (independent variable) and a history of a prior SA (dependent variable) using only age and sex as covariates in a logistic regression model. A higher S-UPPS-P total score (β = 0.05, SE = 0.01, p = 0.008) was associated with prior SA (Fig. 1). Among subscales of the S-UPPS-P (**Supplemental Fig. 1**), higher scores on the negative urgency (β = 0.21, SE = 0.06, p < 0.001) and positive urgency sub-scales (β = 0.15, SE = 0.05, p = 0.002) were also significantly associated with prior SA. Age and sex were not significantly related to a prior SA in any of the models (Table 2).

The results of the tier 2 analysis (**Supplemental Table 2**), which added SSS as covariate, found a significant association between SSS and prior SA in all of the statistical models. In the statistical models, there was still a significant relationship between SA and negative urgency (β = 0.17, SE = 0.17, p = 0.005), positive urgency (β = 0.13, SE = 0.05, p = 0.01), along with a marginally significant relationship (β = 0.04, SE = 0.02, p = 0.052) to S-UPPS-P total score, indicating that participants with a history of previous SA tended to have higher impulsivity scores. Age and sex were again not significant factors in any of the tier 2 analysis models.

To better understand the relationship between impulsivity, SSS, and diagnosis in this study, a fully crossed three-way interaction between impulsivity, diagnosis, and SSS was used in the statistical model in the tier 3 analysis (**Supplemental Table 3**). In the statistical models, a significant three-way interaction (impulsivity × SSS × diagnosis) was observed in the relationship with SA status for S-UPPS-P total score (β = −0.07, SE = 0.03, p = 0.03), negative urgency (β = −0.22, SE = 0.11, p = 0.03), and lack of perseverance (β = −0.27, SE = 0.12, p = 0.03).

**Figure 2:**
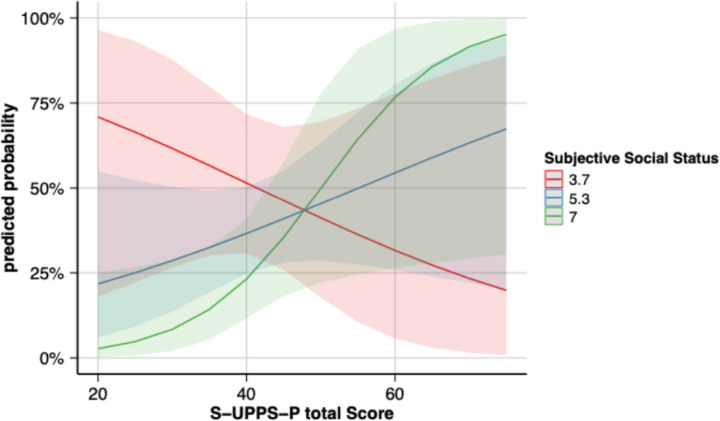
Three-way interaction between total S-UPPS-P score, diagnosis, and SSS. S-UPPS-P = Short UPPS-P Impulsive Behavior Scale.

In Figure 2, sigmoid plots demonstrate the differing relationships in the logistic regression model for SA by diagnosis, SSS, and total impulsivity score. Similar results were seen for negative urgency and lack of perseverance (**Supplemental Figure 2**). In addition to the significant three-way interaction in the statistical models, significant two-way interactions were observed for SSS × Diagnosis and Impulsivity × Diagnosis. A significant two-way interaction for Impulsivity × SSS was also emerged in the model for negative urgency, indicating a stronger association between negative urgency and suicide attempts in those of higher SSS (β = 0.21, SE = 0.09, p = 0.02) and approached significance in the model for S-UPPS-P total score in the same direction (p=0.068). The significant main effect of SSS remained in the models for S-UPPS-P total score (β = −2.33, SE = 1.12, p = 0.04) and negative urgency (β = −2.41, SE = 0.92, p = 0.01). To better understand these interactions, the relationship between SSS and S-UPPS-P total score/subscales are plotted in **Supplemental Figure 3** (total S-UPPS-P score) **and Supplemental Figure 4** (S-UPPS-P Subscales), which raw data points with regression lines are plotted separately for the two groups based on prior history of SA (Attempt+ and Attempt−). The relationship between SSS and S-UPPS-P total score is also plotted in **Supplemental Figure 5** separately for BD and MDD. Finally, diagnosis was significant in the model for total score (β = −15.89, SE = 8.04, p = 0.04), indicating that patients with BD had higher total impulsivity scores compared with those with MDD.

When the subjects BD type II were removed from the statistical models, the pattern of findings and statistical significance remained stable, indicating that the results were not driven by this subgroup.

## 4. Discussion

In this study, we evaluated the relationship between impulsivity as measured by the S-UPPS-P scale and history of prior SA in a cohort of participants with mood disorders (BD and MDD). Our three-tier statistical testing approach revealed that high impulsivity was associated with prior SA, but that this relationship was complex and affected by both SSS and specific mood disorder diagnosis. The findings indicated that impulsivity was more strongly associated with SA among individuals with lower SSS in BD, whereas in MDD the association was stronger among those with higher SSS.

Our tier 1 model results indicated that impulsivity (S-UPPS-P total score, Negative Urgency, and Positive Urgency) was significantly associated with a history of prior SA across participants with any mood disorder. This is in good agreement with a meta-analysis conducted by Bruno et al., where they explored the relationship across the five dimensions of impulsivity based on the UPPS-P model. Their analysis synthesized data from more than 18 prior studies involving both clinical and community samples, with contributions to each of the impulsivity dimensions [65]. The meta-analysis found a significant relationship between each of the dimensions associated with suicide-related outcomes, defined as attempt or ideation, with significantly stronger effect sizes for the relationship between negative and positive urgency as compared to the other dimensions. Furthermore, age and gender were not significant in their moderation analysis. In another study not included in the mentioned meta-analysis, using the Barratt Impulsiveness Scale-11 in a mixed clinical sample, Park et al. observed a significant elevation in trait impulsivity associated with suicidal ideation, suicide attempt, and single versus multiple suicide attempts for overall score as well as the attentional and motor components of the scale [66]. The relationship between trait impulsivity and SA has also been examined in patients with mood disorders, and impulsivity has been found to be a strong predictor of future SA in participants with a diagnosis of BD or MDD [13],[45–46]. Furthermore, elevated impulsivity has also been shown to adversely affect the course of illness in mood disorders by increasing suicide risk [23],[67–68], and mood instability [69]. In individuals with BD, elevated impulsivity during euthymic periods contributes to adverse behaviors such as substance use [70] and poor treatment adherence[71], both of which further increase the likelihood of a future SA.

In our study, individuals with mood disorders and no prior SA reported higher SSS scores compared to those with a history of SA. Furthermore, when a measure of SSS was added into the statistical models used for the tier two analysis, SSS was also significantly associated with prior SA and reduced the amount of variance accounted for by impulsivity. Individuals with lower socioeconomic status (SES) may have fewer economic and psychosocial resources than those with higher SES levels, contributing to lower perceived social status score and inducing a higher prevalence of psychological distress [72], which in turn increases the risk of psychiatric disorders [73]. Conversely, psychiatric conditions including mood disorders are associated with a substantial functional impairment and reduced work productivity, and increased use of health services which leads to even lower financial status [74]. It is also possible that individuals who have attempted suicide have become more isolated (or perceive themselves to be less supported) after the attempt, and thus, they report lower levels of support and more rejection and loneliness [75]. The interplay between low objective social status—which often declines further over the course of illness—and a negatively biased perception of one’s social standing may contribute to poorer clinical outcomes. The findings that SSS was significantly associated with prior SA aligns well with prior studies. A negative association between SSS and suicidal attempts has been observed in various populations, including the elderly [76], adolescents [77–80], patients with MDD [81] and BD [82]. Multiple studies have demonstrated that SSS is inversely associated with depressive symptoms, suicidal ideation, and suicide completion [83]. Research also suggests that SSS may mediate the relationship between SES and both depressive symptoms and suicidality, indicating a significant indirect link between SES and these outcomes through SSS [81],[37]. Additionally, perceived gender discrimination has been linked to SA among adolescents [84], while higher SSS is associated with reduced suicidal ideation in college students [79]. In older adults, the experience of status loss have been correlated with both contemplated and attempted suicide [76]. Longitudinal studies are needed to explore these relationships further and to identify effective interventions for individuals with low SSS and whether these interventions can reduce suicide risk.

In the tier three model, which added diagnosis as well as interaction terms to the model, a significant three-way interaction (Impulsivity × SSS × Diagnosis) was observed. In the BD group, probability of having had prior SA increased more with higher total impulsivity score when SSS was low, but this relationship diminished with increasing SSS. This suggests that in BD, the combination of low SSS and high impulsivity has the greatest association with suicide attempt, whereas higher SSS levels may help to protect against risks conferred by higher impulsivity. In contrast, in the MDD group, the probability of having had prior SA increased with total impulsivity score when SSS was high, and this relationship reversed with decreasing SSS. It is unclear why this pattern would differ from that observed for the BD group. However, it is possible that the underlying circumstances of the suicide attempts in the BD participants could have key differences from those in the MDD group, which would explain a different relationship. For example, if BD participants had more impulsive suicide attempts (perhaps acting with urgency towards a negative emotion and with less pre-planning) than the MDD group, then perhaps it makes sense that high SSS wouldn’t be protective in the MDD group. Indeed, there is some evidence of differences in suicide attempt characteristics between the diagnostic groups, such as higher level of lethality, aggression and impulsivity in patients with bipolar disorder compared with MDD [85–86]. Alternatively, there could be additional information we did not account for that could help explain the different patterns by diagnosis, such as differences in SSS at the time of the suicide attempt or presence of comorbidities. However, it should be noted that the we did not have any participants with a major depression diagnosis who had prior SA with SSS above 7 out of 10, which limits the strength of interpretation for the MDD group results. Future studies with larger sample sizes, greater coverage of the range of SSS, and additional clinical information may be helpful in confirming and clarifying the unexpected pattern in the MDD group.

The findings reported in this study were largely driven by the affective portions of the impulsivity scale: negative and positive urgency. Negative urgency, which is related to the tendency to act rashly when distressed, has been conceptualized as the predictor of both internalizing and externalizing psychopathologies [87]. Internalizing disorders, whose primary symptoms involve internal emotions (e.g., anxiety and depressive states) [88] are well-established risk factors for suicidal behavior [89]. Likewise, externalizing disorders, which involve outwardly directed behaviors (e.g., aggression, impulsivity) [88] are inherently linked to impulsivity and have been associated with increased suicide risk [90–92]. In addition, other externalizing disorders like alcohol use disorder and other substance use disorders have been associated with negative urgency as well as identified as significant risk factors for SA [93]. Positive urgency, on the other hand, is related to the tendency to act impulsively during positive affective states. Interestingly, some evidence suggests that positive urgency is not solely related to positive emotional reactivity, but rather to more complex arrays of emotions, maladaptive behaviors, and a broad range of disorders including substance use and depression [94]. Our results indicate that both emotion-related impulsivity traits—negative and positive urgency—are closely linked to SA. Given the proximity of negative and positive urgency, it may guide clinicians to consider urgency when assessing suicide risk, regardless of its valence. This perspective is supported by Billieux et.al, who argued that differentiating positive and negative urgency as separate constructs is not necessary since they are closely related [95]. Longitudinal studies are still needed to determine more subtle aspects of these associations and their clinical implications.

Despite the clinical relevance of our findings, this study has several limitations. First, we were unable to control for certain demographic, psychological, and clinical variables. Because the YMRS and MADRS scores in our sample were not normally distributed, we did not account for current mood state, which may influence patients’ self-perception of impulsivity and SSS. Given the multi-factorial nature of suicide, other important variables such as a family history of psychiatric disorders [96] or a family history of suicide attempt [97], comorbid medical conditions [98], access to lethal means of suicide [99], recent psychiatric hospitalization [100–101] and history of substance use [102] were not included in our analyses. Second, the cross-sectional nature of the study limits our ability to determine the temporal relationship between impulsivity score, suicidality, and other factors such as SSS. Future longitudinal studies could further clarify the relationship between these measures. Third, the relatively small sample sizes within the MDD and BD groups limited our ability to have precise conclusions about the effect of diagnosis and its interaction with other factors on SA. Future studies with more subjects can lead to a better understanding of the complex interaction of diagnosis, SSS, impulsivity, and suicide. Finally, the sex imbalance in our sample may affect generalizability, as suicide attempts tend to be more prevalent among females [103].

## 5. Conclusion

Our findings support previous findings of a role for impulsivity in SA, which is in turn associated with increased morbidity and mortality in individuals with mood disorders. Furthermore, we observed a significant association between SSS and SA, suggesting that interventions aimed at improving patients’ objective or perceived social status may help reduce suicide risk. Our study also highlights the intricate interplay of multiple factors such as diagnosis, SSS, and impulsivity in influencing SA. Further investigation of these interrelated risk factors is needed for developing more effective, targeted strategies for suicide prevention in mood disorder populations. Notably, impulsivity differed between suicidal and non-suicidal individuals across the entire sample of mood disorders, regardless of diagnostic category. This pattern supports the idea that bipolar disorder and major depressive disorder may exist on a continuum rather than as two completely distinct disorders. Viewing mood disorders dimensionally may help clinicians better understand how impulsivity contributes to suicide risk, particularly in high-risk patients whose behavioral profiles do not fit neatly within traditional diagnostic boundaries.

## Figures and Tables

**Table 1.** Study Demographics Based on Suicide Attempt Status

	*Attempt*+ (n = 66)	*Attempt*− (n = 87)	P-value for Statistical Comparison** *Between Attempt*+ *and Attempt*−
***Sex*** *(M/F)*	19/47	24/63	*0.98*
***Age*** *	40.4 ± 14.9	36.5 ± 14.4	0.13
***Education*** *	15.4 ± 2.4	15.1 ± 2	0.38
***MADRS*** *	17.5 ± 11.4	12.2 ± 7.4	0.001
***YMRS*** *	7.4 ± 8.2	3.4 ± 3.8	0.002
***ACE*** *	4.2 ± 2.4	2.5 ± 2.1	< 0.001
***SSS*** *	4.9 ± 1.7	5.7 ± 1.5	0.001
Diagnosis*** *(MDD/BD)*	26/40	53/34	0.01

* Values reported as mean± standard deviation

** MDD=Major Depressive Disorder, BD=Bipolar Disorder.

*** Chi-squared test for sex and diagnosis while all other comparisons used t-test.

**Table 2.** Comparison of S-UPPS-P total score and subscales between suicide attempters and non-attempters, **Tier 1 Analysis**.

Model Terms								
S-UPPSP	Intercept		Impulsivity		Age		Sex	
	B* (SE)	p-value	B* (SE)	p-value	B* (SE)	p-value	B* (SE)	p-value
***Total Score***	− 3.17 (1.03)	0.002**	0.05 (0.02)	0.008**	0.02 (0.01)	0.14	− 0.02 (0.37)	0.95
***Negative Urgency***	− 3.02 (0.83)	<0.001**	0.21 (0.06)	<0.001**	0.01 (0.01)	0.20	0.07 (0.38)	0.85
***Lack of Perseverance***	− 0.03 (0.77)	0.96	− 0.09 (0.07)	0.23	0.01 (0.01)	0.27	− 0.03 (0.37)	0.91
***Lack of Premeditation***	− 0.82 (0.84)	0.33	0.006 (0.08)	0.94	0.01 (0.01)	0.25	0.025 (0.36)	0.94
***Sensation Seeking***	− 1.22 (0.74)	0.09	0.04 (0.05)	0.42	0.01 (0.01)	0.20	− 0.06 (0.38)	0.88
***Positive Urgency***	− 2.13 (0.66)	0.001**	0.15 (0.05)	0.002**	0.01 (0.01)	0.20	− 0.02 (0.37)	0.95

* All B coefficients represent unstandardized estimates, reflecting the change in log-odds per one-unit increase in the predictor.

** Statistically significant model parameters (p<0.05)

## Data Availability

Data is available on the national data archive (NDA) collection ID 4549.
